# PASCAL versus MitraClip-XTR edge-to-edge device for the treatment of tricuspid regurgitation: a propensity-matched analysis

**DOI:** 10.1007/s00392-020-01784-w

**Published:** 2020-12-12

**Authors:** Atsushi Sugiura, Johanna Vogelhuber, Can Öztürk, Zita Schwaibold, David Reckers, Tadahiro Goto, Refik Kavsur, Marc Ulrich Becher, Sebastian Zimmer, Georg Nickenig, Marcel Weber

**Affiliations:** 1 Herzzentrum Bonn, Medizinische Klinik und Poliklinik II, Universitätsklinikum Bonn, Venusberg-Campus 1, 53127 Bonn, Germany; 2 Department of Clinical Epidemiology and Health Economics, School of Public Health, University of Tokyo, TXP Medical Co. Ltd., Tokyo, Japan

**Keywords:** Transcathether, Edge-to-edge, Tricuspid regurgitation, MitraClip, PASCAL, Propensity score matching

## Abstract

**Background:**

Transcatheter tricuspid valve repair (TTVR) is a promising technique for the treatment of tricuspid regurgitation (TR). Data comparing the performance of novel edge-to-edge devices (PASCAL and MitraClip-XTR) are scarce.

**Methods:**

We identified 80 consecutive patients who underwent TTVR using either the PASCAL or MitraClip-XTR system to treat symptomatic TR from July 2018 to June 2020. To adjust for baseline imbalances, we performed a propensity score (PS) 1:1 matching. The primary endpoint was a reduction in TR severity by at least one grade at 30 days.

**Results:**

The PS-matched cohort (*n* = 44) was at high-surgical risk (EuroSCORE II: 7.5% [interquartile range (IQR) 4.8–12.1%]) with a mean TR grade of 4.3 ± 0.8 and median coaptation gap of 6.2 mm [IQR 3.2–9.1 mm]. The primary endpoint was similarly observed in both groups (PASCAL: 91% vs. MitraClip-XTR: 96%). Multiple device implantation was the most common form (59% vs. 82%, *p* = 0.19), and the occurrence of SLDA was comparable between the PASCAL and MitraClip-XTR system (5.7% [2 of 35 implanted devices] vs. 4.4% [2 of 45 implanted devices], *p* = 0.99). No periprocedural death or conversions to surgery occurred, and 30-day mortality (5.0% vs. 5.0%, log-rank *p* = 0.99) and 3-month mortality (10.0% vs. 5.0%, log-rank *p* = 0.56) were similar between both groups. During follow-up, functional NYHA class, 6-min walking distance, and health status improved in both groups.

**Conclusions:**

Both TTVR devices, PASCAL and MitraClip-XTR, appeared feasible and comparable for an effective TR reduction. Randomized head-to-head comparisons will help to further define the appropriate scope of application of each system.

**Supplementary Information:**

The online version contains supplementary material available at 10.1007/s00392-020-01784-w.

## Background

Tricuspid regurgitation (TR) is no longer called “a forgotten valvular disease.” Instead, TR has turned out as a prognostic devastator. Previous cohort studies demonstrated a clear association between significant TR and excess mortality and reduced quality of life (QOL) [1, 2]. Due to the high-surgical risk in this population, a catheter-based and minimally invasive procedure is thought to be promising to reduce TR safe and effective without excess periprocedural risk. Recent encouraging results of transcatheter tricuspid valve repair (TTVR) have been observed with different technologies [3–6], and transcatheter edge-to-edge repair is the most prevalent technique for leaflet approximation [7]. However, previous cohort studies with the MitraClip device suggested that a more severe TR and longer coaptation gap are associated with an increased risk of the suboptimal TR reduction [8, 9]. The concern was mitigated using the novel devices (i.e., PASCAL and MitraClip-XTR systems) with extended device arms, which show a higher rate of implant success [5, 10]. The PASCAL system also offers wide paddles, a central spacer, and an optional independent clasping [11]. Despite the high demand for these new technologies, data comparing the performance of the PASCAL versus MitralClip-XTR systems are absent.

In this context, we aimed (1) to compare the PASCAL and MitralClip-XTR system concerning the efficacy of TR reduction, and (2) to assess clinical outcomes after the procedures.

## Methods

### Study population

The study was designed as a retrospective analysis of data from the Bonn registry, which is a single-center, prospective, consecutive database of patients treated at the University of Bonn Heart Center. We identified consecutive patients who underwent a TTVR using the PASCAL or MitraClip-XTR systems from April 2018 to June 2020. The MitraClip-XTR system was utilized as an off-label use. The PASCAL system was available from February 2019 (off-label use) and commercially available since May 2020. All patients had symptomatic TR and were considered as inoperable or at high-surgical risk. After a standardized diagnostic workup including transesophageal echocardiography (TEE), the decision to perform the intervention was taken by the interdisciplinary heart team of the Heart Center Bonn. We excluded patients who underwent a combination of edge-to-edge and annuloplasty technique. This study was approved by the ethics committee of the individual center and was conducted in accordance with the Declaration of Helsinki. All patients participated in the study after written informed consent was obtained.

### Procedure

Procedures were performed under general anesthesia with 2D- and 3D-TEE and fluoroscopic guidance. Both the PASCAL and MitraClip-XTR system have been well described previously [3, 5]. After device placement, the acute reduction in TR was quantified. The discretion if a second or third device had to be used was left to the treating physicians.

### Echocardiographic parameters

We assessed echocardiographic parameters performed at baseline, 30 days, and follow-up [9]. TEE was performed at baseline and during the procedure with a Vivid E95 ultrasound system (GE health care, GE Healthcare, Illinois, USA). The severity of TR was graded as follows: grade 0, none; 1 +, mild; 2 +, moderate; 3 + severe; 4 + massive; 5 +, torrential, in which qualitative measurements were assessed as best as was possible [12–14]. All measurements were reviewed by two independent cardiologists dedicated to echocardiographic evaluation.

### Outcomes

The primary endpoint was a reduction in TR severity by at least one grade at 30 days. Secondary endpoints were implant success during the procedure, 30-day mortality, and 3-month mortality. Implant success was defined as successful delivery and deployment of one or more clips to achieve leaflet approximation and retrieval of the delivery system [9]. Clinical outcomes were prospectively assessed during scheduled hospital visits. Telephone interviews were also performed with the patients’ general practitioners or family. Symptomatic capacity, such as New York Heart Association (NYHA) functional class and 6-min walking distance and the Medical Outcomes Study Short-Form (SF-36) Health Survey, was also evaluated [15]. The SF-36 is composed of physical summary scores (SF-36 PCS) and mental summary scores (SF-36 MCS), with an overall population mean of 50 and SD of 10, in which higher scores indicate better health status. These symptomatic and health status were prospectively assessed at baseline and follow-up.

### Statistical analysis

Categorized variables are presented as numbers and percentages. Normally distributed variables are presented as mean ± standard deviation and compared using t tests. In contrast, non-normal distributed variables are reported as medians and interquartile ranges (IQRs) and compared using the Mann–Whitney *U* test. A paired *t* test was used to compare a series of variables with regard to symptomatic functional capacity (i.e., 6MWD and 36-SF).

Propensity score (PS) was calculated for each patient using multivariate logistic regression that estimates the propensity toward belonging to a specific treatment group (PASCAL versus MitraClip-XTR). This was performed using multivariable logistic regression. Following covariates were included in this PS model: right atrial area, tricuspid annulus diameter, coaptation gap, effective regurgitant orifice area (EROA), vena contracta, TR jet location, and TR grade [5, 9]. We conducted one-to-one matching based on the PS with the nearest-neighbor algorithm method. Standardized differences were reported for baseline characteristics. After PS matching, we compared procedural and clinical outcomes between PASCAL and MitraClip-XTR groups.

In the sensitivity analysis, we used stabilized inverse probability weighting (stabilized IPW) to examine the association of PASCAL with outcomes in this observational study [16]. Weighting subjects by the inverse probability to have an exposure (PASCAL) creates a synthetic sample in which the exposure is independent of measured baseline covariates. Although the conventional IPW enables us to obtain unbiased estimates of the exposure's effect on each outcome, subjects with a very low or high propensity score can increase the variability of the estimated effects. Stabilized IPW addresses this issue and directly estimates both the main effect and its variance from conventional regression models.

Two-tailed *p* values < 0.05 were regarded as statistically significant. All statistical analyses were performed using EZR version 1.37 (Saitama Medical Center, Jichi Medical University, Saitama, Japan) or R version 3.5.2 (R Foundation for Statistical Computing).

## Results

A total of 80 patients underwent TTVR with the PASCAL system (*n* = 22) or MitraClip-XTR (*n* = 58) during the study period and were included in the analysis (Supplemental Fig. 1). Baseline characteristics before matching are shown in Supplemental Table 1. Overall, the study patients were mean 78 years old and predominantly female (58%), highly symptomatic (NYHA functional class III or IV: 93%) and had a significant burden of comorbidities (coronary artery disease: 60%, atrial fibrillation: 94%, and history of cardiac surgery: 64%), which translated into high-surgical risk (EuroSCORE II: 8.3% [IQR 5.3, 12.6%].

Patients treated with the PASCAL system had a larger right atrial area (39.7 mm^2^ [IQR 35.4, 42.6 mm^2^] vs. 30.3 mm^2^ [25.3, 37.8 mm^2^], *p* = 0.005) and a greater TR (4.3 ± 0.8 vs. 3.8 ± 0.9, *p* = 0.02) with a larger EROA (74.5 mm^2^ [51.5, 119.8 mm^2^] vs. 46.0 mm^2^ [34.0, 64.5 mm^2^], *p* < 0.001) compared to those treated with the MitraClip-XTR system. Furthermore, the PASCAL group showed more often TR in the antero-posterior commissure compared to the MitraClip-XTR group (36% vs. 12%, *p* = 0.02).

After PS matching, 22 pairs of matched patients were identified. Baseline characteristics are presented in Table 1. The PS-matched cohort was at high-surgical risk (EuroSCORE II: 7.5% [IQR 4.8, 12.1%]), with a median EROA of 71.5 mm^2^ [IQR 54.5, 110.3 mm^2^] and coaptation gap of 6.2 mm [IQR 3.2, 9.1 mm]. The baseline characteristics were comparable between the groups, including age, sex, NYHA functional class, EuroSCORE II, left-ventricular ejection fraction, concomitant MR, right atrial area, tricuspid annulus diameter, coaptation gap, EROA, vena contracta, regurgitant volume, and TR jet location.

**Table 1:** Baseline characteristics after PS matching

	All (*n* = 44)	Pascal (*n* = 22)	MitraClip-XTR (*n* = 22)	Standardized difference	*p* value
Age (year)	79 ± 6	79 ± 5	78 ± 7	0.22	0.74
Sex female [*n* (%)]	28 (64)	14 (64)	14 (64)	0.00	0.99
BMI (kg/m^2^)	24.9 [21.4, 27.4]	24.5 [20.8, 27.4]	25.3 [22.3, 27.3]	0.25	0.83
Hypertension[*n* (%)]	37 (84)	18 (82)	19 (86)	0.002	0.99
Diabetes mellitus [*n* (%)]	9 (20)	4 (18)	5 (23)	0.04	0.99
COPD [*n* (%)]	10 (23)	7 (32)	3 (14)	0.14	0.28
Atrial fibrillation [*n* (%)]	41 (93)	21 (96)	20 (91)	0.001	0.99
Coronary artery disease [*n* (%)]	24 (55)	13 (59)	11 (50)	0.01	0.76
Prior cardiac surgery [*n* (%)]	30 (68)	14 (64)	16 (73)	0.005	0.75
Prior pacemaker/ICD/CRT implantation [*n* (%)]	11 (25)	5 (23)	6 (27)	0.02	0.99
NYHA III/IV [*n* (%)]	41 (93)	21 (96)	20 (91)	0.001	0.99
EuroSCORE II (%)	7.5 [4.8, 12.1]	7.8 [4.3, 12.1]	7.4 [6.2, 11.6]	0.20	0.67
NT-pro-BNP (pg/ml)	2039 [1241, 3087]	1892 [1150, 3290]	2254 [1396, 3470]	0.12	0.17
e-GFR (ml/min/1.73m^2^)	49.7 [39.2, 66.1]	46.8 [35.4, 64.9]	52.6 [40.9, 67.0]	0.21	0.54
Total bilirubin (mg/dl)	0.8 [0.6, 1.1]	0.8 [0.5, 1.0]	0.9 [0.7, 1.1]	0.19	0.41
AST (IU/L)	29.0 [22.0, 34.0]	24.0 [22.0, 32.0]	32.5 [25.8, 37.0]	0.54	0.08
Hemoglobin (g/dl)	12.0 [10.5, 13.0]	11.9 [10.8, 13.0]	12.2 [10.5, 13.1]	0.06	0.84
LVEF (%)	57.0 [55.0, 62.1]	57.0 [55.2, 63.9]	56.9 [55.0, 58.9]	0.27	0.37
MR moderate to severe or more [*n* (%)]	5 (11)	3 (14)	2 (9)	0.16	0.99
TR massive or torrential [*n* (%)]	32 (73)	16 (73)	16 (73)	0.00	0.99
TR grade	4.3 ± 0.8	4.3 ± 0.8	4.2 ± 0.9	0.11	0.72
EROA (mm^2^)	71.5 [54.5, 110.3]	74.5 [51.5, 119.8]	70.5 [58.5, 88.8]	0.24	0.43
Vena contracta (mm)	9.5 [7.2, 12.3]	9.6 [7.2, 11.8]	9.0 [7.1, 12.5]	0.13	0.66
Regurgitant volume (ml)	51.8 [45.3, 68.3]	51.8 [43.8, 70.1]	53.6 [45.6, 64.0]	0.01	0.97
TR jet location					
Central or antero-septal commissure	44 (100)	22 (100)	22 (100)	0.00	0.99
Postero-septal commissure	38 (86)	19 (86)	19 (86)	0.00	0.99
Antero-posterior commissure	15 (34)	8 (36)	7 (32)	0.01	0.99
Coaptation gap (mm)	6.2 [3.2, 9.1]	6.2 [3.3, 9.2]	6.6 [3.8, 9.0]	0.08	0.78
Tricuspid annulus diameter (mm)	44.5 [40.0, 49.8]	45.0 [42.0, 49.0]	40.5 [39.0, 51.0]	0.42	0.17
RA area (mm^2^)	36.5 [29.5, 41.8]	39.7 [35.4, 42.6]	32.4 [28.0, 39.3]	0.54	0.08
RV diameter (mm)	45.8 [31.0, 50.5]	54.0 [46.3, 57.8]	49.0 [45.3, 53.5]	0.32	0.30
RV diameter mid (mm)	37.5 [31.8, 46.0]	39.5 [35.3, 48.5]	36.5 [29.5, 40.5]	0.49	0.11
TAPSE (mm)	17 [15.0, 20.5]	16.5 [12.5, 19.0]	18.0 [16.0, 22.0]	0.17	0.14

*BMI* body mass index, *COPD* chronic obstructive pulmonary disease, *CRT* cardiac resynchronization therapy, *EROA* effective orifice regurgitant area, *ICD* intracardiac defibrillator, *GFR* glomerular filtration ratio, *MR* mitral regurgitation, *LVEF* left-ventricular ejection fraction, *NT-pro-BNP* NT-pro-brain natriuretic peptide, *RA* right atrial, *RV* right ventricular, *TAPSE* tricuspid annular plane systolic excursion, *TR* tricuspid regurgitation

### Periprocedural findings

**Fig. 1:**
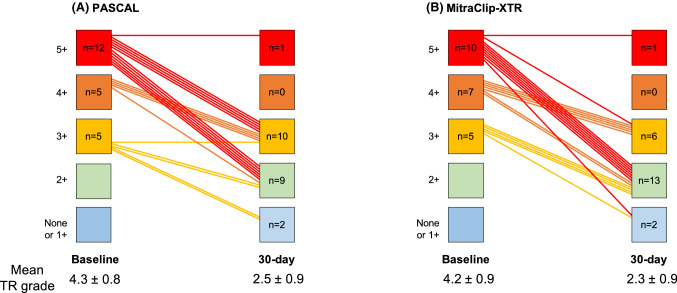
Change in the grade of tricuspid regurgitation. Shown are changes in the severity of tricuspid regurgitation **a** in the PASCAL and **b** the MitraClip-XTR group. Each group showed a significant reduction in tricuspid regurgitation from baseline to 30-day follow-up. *N* number of patients

Procedural findings in the overall cohort are summarized in Supplemental Table 2, and those in the PS-matched cohort are shown in Table 2. In the PS-matched cohort, implantation success was achieved in 20 (91%) patients in the PASCAL group and 21 (96%) in the MitraClip-XTR group. Both groups showed a significant reduction in TR (PASCAL: grade 4.3 ± 0.8 to 2.5 ± 0.9, *p* < 0.001; MitraClip-XTR: grade 4.2 ± 0.9 to 2.3 ± 0.9, *p* < 0.001: Fig. 1) with a similar rate of the primary endpoint between the groups (PASCAL: 91% vs. MitraClip-XTR: 96%). Consequently, TR ≤ 2 + at 30 days was observed in 11 (50%) patients in the PASCAL group and 15 (68%) patients in the MitraClip-XTR group (*p* = 0.56). Devices were implanted mainly in the antero-septal commissure in both groups, PASCAL and MitraClip-XTR (80% [28 of 35 implanted devices] vs. 80% [36 of 45 implanted devices], *p* = 0.99), followed by postero-septal position. Multiple device implantation (≥ 2) was the most common strategy for both PASCAL and MitraClip-XTR systems (59% [13 of 22 patients] vs. 82% [18 of 22 patients], *p* = 0.19). On average, the number of devices was significantly lower in the PASAL group (1.6 ± 0.8 vs. 2.0 ± 0.7, *p* = 0.04). The independent clasping was applied in 19 (86%) patients in the PASCAL group. The occurrence of SLDA was similar between the groups (5.7% [2 of 35 implanted devices] vs. 4.4% [2 of 45 implanted devices], *p* = 0.99). No surgical conversion or periprocedural mortality occurred.

**Table 2:** Procedural findings

	All (*n* = 44)	Pascal (*n* = 22)	MitraClip-XTR (*n* = 22)	*p* value
Device successfully deployed [*n* (%)]	41 (93)	20 (91)	21 (96)	0.99
TR reduction at least 1 + [*n* (%)]	41 (93)	20 (91)	21 (96)	0.99
Number of devices implanted [*n* (%)]				0.29
0	2 (5)	2 (9)	0 (0)	
1	11 (25)	7 (32)	4 (18)	
2	24 (54)	11 (50)	13 (59)	
3	7 (16)	2 (9)	5 (23)	
Devices per patient (devices in total/number of patients)	1.8 ± 0.8 (80/44)	1.6 ± 0.8 (35/22)	2.0 ± 0.7 (45/22)	0.04
Implantation site of devices^a^				0.99
Antero-septal commissure	54 (80)	28 (80)	36 (80)	
Postero-septal commissure	16 (20)	7 (20)	9 (20)	
Antero-septal commissure	0	0	0	
Independent clasping [*n* (%)]	19 (43)	19 (86)	NA	NA
Single leaflet device attachment [*n* (%)]	2 (9)	2 (9)	2 (9)	0.99
Procedure time (min)	62.0 [52.0, 98.0]	62.0 [52.5, 90.0]	67.0 [45.3, 97.8]	0.99
Periprocedural death [*n* (%)]	0	0	0	0.99
Conversion to surgery [*n* (%)]	0	0	0	0.99
Pericardial tamponade [*n* (%)]	3 (7)	2 (9)	1 (5)	0.99
Major bleeding [*n* (%)]	3 (7)	2 (9)	1 (5)	0.99
Multiple blood transfusion [*n* (%)]	4 (9)	2 (9)	2 (9)	0.99
Stroke	0	0	0	0.99
Post-procedural mean TV gradient (mmHg)	2.5 ± 1.1	2.5 ± 0.9	2.5 ± 1.2	0.88
Post-procedural TR grades	2.4 ± 0.9	2.5 ± 0.9	2.3 ± 0.9	0.41
Mean grade of TR reduction	1.9 ± 1.0	1.8 ± 1.0	2.0 ± 1.1	0.66

*TR* tricuspid regurgitation, *TV* tricuspid valve

^a^Numbers and percentages indicate number/percentage of clips implanted

In the sensitivity analysis (i.e., stabilized IPW), similar to the primary findings using PS matching, there were no significant differences between the PASCAL and MitraClip-XTR groups in the primary outcome (OR 0.85, 95% CI 0.12–5.85, *p* = 0.87) or successful implantation (OR 1.05, 95% CI 0.15–7.62, *p* = 0.96).

Two patients had torrential TR after the treatment. In one patient treated with the PASCAL system, implantation was not feasible due to a pronounced coaptation gap. In one patient treated with the MitraClip-XTR system, the second device was tangled with chordae—although the first device could previously been deployed successfully. After retracting the system, color-Doppler echocardiography showed torrential TR due to leaflet prolapse and chordae rupture. These patients were managed conservatively with optimal medical therapy.

### Clinical outcome

**Fig. 2:**
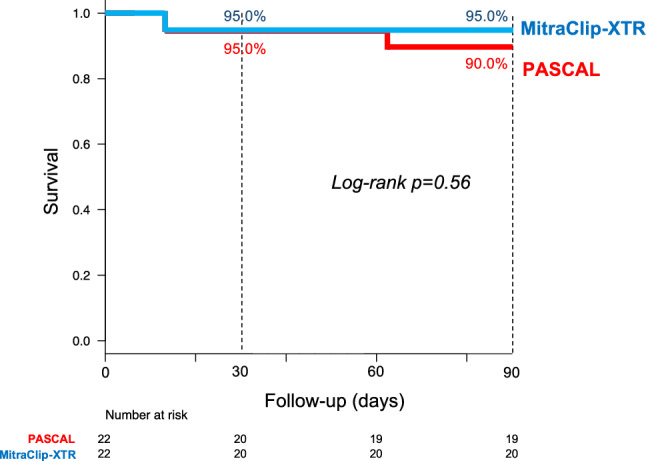
Survival analysis after procedure. Three-month mortality was similar between the PASCAL and the MitraClip-XTR groups in the propensity-matched cohort (10.0% vs. 5.0%, log-rank *p* = 0.56)

The 3-month follow-up was completed in 68 (85%) patients in the overall cohort and 36 (82%) patients in the PS-matched cohort. Survival curves are depicted for the overall cohort in Supplemental Fig. 2 and for the PS-matched cohort in Fig. 2. In the PS-matched cohort, with a median follow-up period of 5.0 months (IQR 3.1, 9.7 months), three patients had died during the first 3 months. There were no significant difference in 30-day (5.0% vs. 5.0%, log-rank *p* = 0.99) or 3-month mortality between both groups (10.0% vs. 5.0%, log-rank *p* = 0.56). Similarly, in the sensitivity analysis, there were no significant differences between the groups in the 30-day mortality (OR 0.92, 95% CI 0.09–9.89, *p* = 0.95) or 3-month mortality (OR 0.89, 95% CI 0.16–5.03, *p* = 0.89).

**Fig. 3:**
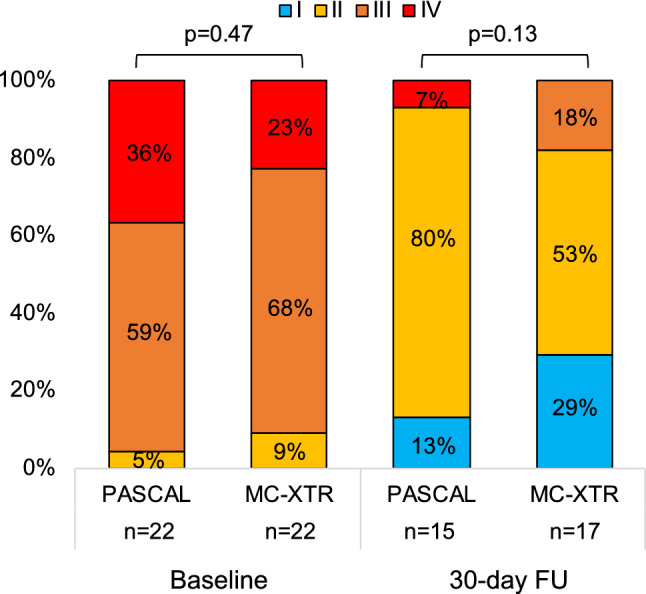
NYHA functional class at baseline and follow-up. NYHA functional class improved in the PASCAL group (NYHA I/II: 5% at baseline to 93% at follow-up) and in the MitraClip-XTR group (NYHA I/II: 13% to 82%). *FU* follow-up, *NYHA* New York Heart Association

**Fig. 4:**
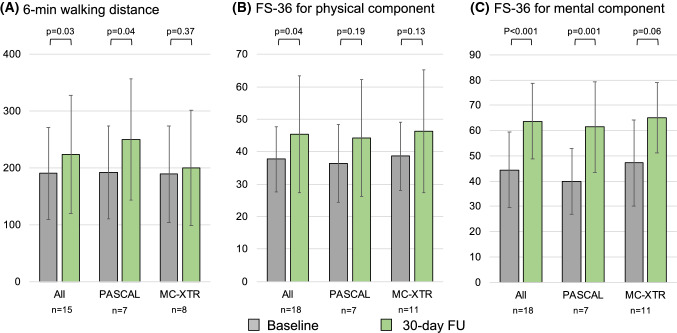
Six-min walk distance test and quality-of-life measures. Shown are changes in 6-min walking distance and health status for the physical (SF-36 PCS) and mental (SF-36 MCS) components. Overall, there was a significant improvement of 6-min walking distance and health status from baseline to follow-up. Trends toward the improvement of these variables were seen in both groups. *SF-36 PCS* Short-Form 36 Health Survey physical summary score, *SF-35 MCS* Short-Form 36 Health Survey mental summary score

With regard to the symptomatic status, functional NYHA class showed a significant improvement in both groups, with the percentage of NYHA class I/II increasing from 5% at baseline to 93% at follow-up in the PASCAL group and 13–82% in the MitraClip-XTR group (Fig. 3). Figure 4 represents the change in 6-min walking distance and health status assessed by the SF-36. Overall, 6-min walking distance improved from 190.6 ± 81.2 m at baseline to 223.5 ± 104.1 m at follow-up (*p* = 0.03). Similarly, there was a significant improvement of health status for the physical component (SF-36 PCS: 37.7 ± 10.9 to 45.4 ± 18.1, *p* = 0.04) and the mental component (SF-36 MCS: 44.4 ± 15.9 to 63.7 ± 15.8, *p* < 0.001) from baseline to follow-up. With a limited sample size, trends toward an improvement of 6-min walking distance and health status were observed in both groups.

## Discussion

Up to now, leaflet approximation devices for edge-to-edge repair are most frequently used for transcatheter tricuspid valve reconstruction. By now, the two devices, PASCAL and TriClip, are CE certified. Although it is not a perfect repair, other options like annuloplasty or transcatheter valve replacement are either complex or still in the early stages of clinical investigation.

In this propensity score matched comparison of the two available edge-to-edge repair techniques, we found that both devices—PASCAL and MitraClip-XTR—showed similar acute procedural success rates with proven feasibility, safety, and a significant reduction in TR after TTVR. Furthermore, after both types of edge-to-edge repair, clinical outcomes and quality of life were comparable and in both groups promising.

Successful TR reduction translates into lower mortality. Besler et al. and Orban et al. have reported that successful transcatheter TR reduction was associated with a reduced risk of mortality [8, 17]. However, since July 2018 when the MitraClip-XTR device first got commercially available and since February 2019 when the Edwards mitral PASCAL was released, there has been limited experience with these device systems reported and published. There are only a few publications accessible for off-label use of MitraClip-XTR in a tricuspid position [10, 18, 19] and one for PASCAL in a tricuspid position [5], which serve as a basis for comparison. The procedural success rate in our study (93% in the PS-matched cohort) was comparatively high, as Braun et al. [10] reported 87% in 31 XTR patients, and Fam et al. [5] showed a success rate of 86% in 28 Pa patients. TR reduction by ≥ 1 grade until discharge was achieved in > 90% in both groups of the PS-matched cohort. For Triluminate—the prospective single-arm multicenter trial for the TriClip System (NT size)—a TR reduction by ≥ 1 grade was obtained in 91% of patients [9]. In both the Triluminate trial and our analysis, a five-class grading scheme was used to assess the severity of TR [12], as massive or torrential TR, which are included in the five-class grading, are associated with a higher risk of death and readmission of HF [20]. Braun and Fam et al. did not report TR reduction at discharge, but indicated a TR reduction to grade 2 + or lower in 85% of PASCAL cases and 69% for XTR patients. TR reduction of the discussed studies is not comparable as the authors of the XTR report did not apply the five-scale grading scheme.

Another essential parameter for safety is 30-day mortality. Whereas a 30-day mortality of 5.0% in both groups of our cohort seemed encouraging, the Triluminate trial accounted for 0% mortality at 30-day follow-up [9]. Fam et al. reported a 30-day mortality of 7.1% [5]. For a better comparison, baseline risk scores of different studies have to be mentioned, which all range in a moderately-to-highly elevated operation risk—estimated by EuroSCORE II—between 6.2 (PASCAL report by Fam et al.) and 8.6% (Triluminate cohort) [5, 9].

Effective and durable reduction of TR is another crucial therapeutic objective of TTVR. We observed a TR reduction by ≥ 1 grade in 93% of patients in the PS-matched cohort, whereas 86% were reported for the Triluminate trial. However, other groups investigating the performance of XTR [10] or PASCAL [5] rather stated the percentage of reduction to grade 2 + or less at 30-day follow-up (69–85%). Interestingly, the Triluminate trial showed 56% of patients at TR grade 1 + or 2 + after 30 days with the smaller NT device and not the XTR clip. We saw a 50% reduction to grade 2 + or less for PASCAL and 68% for XTR after 30 days without any significance between groups (*p* = 0.56). A potential explanation for different TR reduction could be SLDA. However, SLDA did not turn out to be pronounced in both groups with each 9%, whereas the earlier studies reported the rates of nearly 7% [5, 9]. Another explanation for inacceptable TR reduction could be the gap widths. Although patients with larger coaptation gaps could be treated with both devices, the implantation and procedural success rates would probably be lower [8, 9]. Our PS matching aimed to create two almost equal cohorts to overcome selection bias and any anatomic imbalance. Our matched groups showed a similar safety and efficacy profile, despite lacking the optional independent clasping in the MitraClip-XTR system. One explanation could be related to acute tricuspid valve remodeling after clipping. Annular diameter reduction and pulling up leaflets caused by a first clip can facilitate to deploy the second clip correctly [21]. The present study is—to our knowledge—the first PS-matched analysis comparing these two new treatment options. We have to point out that the mitral PASCAL is identical to the newly CE-marked tricuspid device, whereas the recently CE-marked TriClip device has besides its identical clip some iterations on the septo-lateral steering mechanism and the distal curve of the guiding catheter.

Clinical outcome and NYHA functional scale after both types of edge-to-edge repair were promising and comparable to previously published data of both devices that reported 69–85% of patients in NYHA class I/II after 30 days [5, 10, 22]. Coupled with the improvement of NYHA, the amelioration of the 6-min walk distance and the SF-36 questionnaires support our findings' validity.

### Limitations

Several limitations should be acknowledged. First, the present study was conducted retrospectively, based on a single-center and relatively small sample size cohort. Therefore, a certain patient selection bias might have impacted our results, and the analysis might be underpowered. Nevertheless, we used PS-matching analysis to overcome selection bias and any anatomic imbalance. In turn, the rate of successful TR reduction was consistently observed in the PASCAL and MitraClip groups. Assuming that successful TR reduction is a significant predictor of mortality [8, 17], the comparable results regarding 30-day and 3-month mortality between the groups may be conceivable. In addition, the improvements in the functional capacity, which were observed consistently in both groups, may prove the validity of our findings. Second, clinical outcomes and echocardiographic findings were not adjudicated by a central Core-Lab. Third, although the main concept of both systems is identical (edge-to-edge repair), the difference regarding the learning curve in each system might affect the procedural outcomes. The MitraClip System (NTR device) has been utilized for TTVR since 2015, while the PASCAL system is available since November 2018. Further studies should aim to validate our findings.

## Conclusions

In this PS-matched cohort, we found that edge-to-edge treatment with either the PASCAL or the MitraClip-XTR device was feasible, effective, and safe in patients with severe tricuspid regurgitation and was associated with clinical improvement. Whether TR reduction is associated with improvement in relevant clinical outcomes parameters such as mortality and rehospitalization has to be answered in future studies. Randomized trials investigating the effect for both devices compared to optimal medical treatment on reducing TR are ongoing (Triluminate Pivotal Trial, NCT: 03,904,147; Edwards PASCAL Transcatheter Valve Repair System Pivotal Clinical Trial, NCT: 04,097,145). Even more substantial would be a randomized head-to-head comparison between these two TTVR devices and optimal medical therapy.

## Supplementary Information

Below is the link to the electronic supplementary material.

Supplementary file2 (DOCX 15 KB)

Supplementary file3 (DOCX 17 KB)

Supplemental Figure 1. Study population. During the study period, 80 patients were treated with the PASCAL or MitraClip-XTR systems for the treatment of TR. After propensity score matching, 44 patients (22 PASCAL vs. 22 MitraClip-XTR) were included into the present analysis

Supplemental Figure 2. Survival analysis for entire cohort. There was no significant difference in 30-day and 3-month mortality between the PASCAL and MitraClip-XTR groups

Supplementary file1 (XLSX 186 KB)
