# Supplementary material for: PASCAL versus MitraClip-XTR edge-to-edge device for the treatment of tricuspid regurgitation: a propensity-matched analysis
Source: Clin Res Cardiol. 2020 Dec 12;110(3):451–9. doi: 10.1007/s00392-020-01784-w (PMC7907034; doi:10.1007/s00392-020-01784-w)
Supplement: Supplementary file 1 — Supplementary file2 (DOCX 15 KB) [file 392_2020_1784_MOESM1_ESM.docx]

**Supplemental Table 2. Procedural findings in overall cohort**

|  | All  n=80 | Pascal  n=22 | MitraClipXTR  n=58 | p value |
| --- | --- | --- | --- | --- |
| Device successfully deployed, n (%) | 74 (93) | 20 (91) | 54 (93) | 0.67 |
| TR reduction at least 1+, n (%) | 73 (91) | 20 (91) | 53 (91) | 0.99 |
| Number of devices implanted, n (%) |  |  |  | 0.81 |
| 0 | 5 (6) | 2 (9) | 3 (5) |  |
| 1 | 23 (29) | 7 (32) | 16 (28) |  |
| 2 | 44 (55) | 11 (50) | 33 (57) |  |
| 3 | 8 (10) | 2 (9) | 6 (10) |  |
| Devices per patient (devices in total / number of patients) | 1.7 ± 0.7 (135/80) | 1.6 ± 0.8 (35/22) | 1.7 ± 0.7 (100/58) | 0.48 |
| Implantation site of devices* |  |  |  | 0.46 |
| Antero-septal commissure | 112 (83) | 28 (80) | 84 (84) |  |
| Postero-septal commissure | 23 (17) | 7 (20) | 16 (16) |  |
| Antero-septal commissure | 0 | 0 | 0 |  |
| Independent clasping, n (%) | 19 (24) | 19 (86) | NA | NA |
| Single leaflet device attachment, n (%) | (8) | 2 (9) | 4 (7) | 0.39 |
| Procedure time (min) | 73.0 [52.5, 95.5] | 62.0 [52.5, 90.0] | 77.0 [58.3, 95.3] | 0.54 |
| Periprocedural death, n (%) | 0 | 0 | 0 | 0.99 |
| Conversion to surgery, n (%) | 0 | 0 | 0 | 0.99 |
| Pericardial tamponade, n (%) | 3 (4) | 2 (9) | 1 (2) | 0.99 |
| Major bleeding, n (%) | 4 (5) | 2 (9) | 2 (4) | 0.31 |
| Multiple blood transfusion, n (%) | 3 (6) | 2 (9) | 3 (5) | 0.61 |
| Stroke, n (%) | 0 | 0 | 0 | 0.99 |
| Post-procedural mean TV gradient (mmHg) | 2.2 [1.7, 2.9] | 2.6 [1.8, 3.1] | 2.1 [1.7, 2.9] | 0.75 |
| Post-procedural TR grades | 2.2 ± 0.9 | 2.5 ± 0.9 | 2.1 ± 0.9 | 0.09 |
| Mean grade of TR reduction | 1.8 ± 1.0 | 1.8 ± 1.0 | 1.8 ± 1.1 | 0.82 |

*: Numbers and percentages indicate number / percentage of clips implanted.

**Abbreviations**: TR, tricuspid regurgitation; TV, tricuspid valve.
