# Supplementary material for: PASCAL versus MitraClip-XTR edge-to-edge device for the treatment of tricuspid regurgitation: a propensity-matched analysis
Source: Clin Res Cardiol. 2020 Dec 12;110(3):451–9. doi: 10.1007/s00392-020-01784-w (PMC7907034; doi:10.1007/s00392-020-01784-w)
Supplement: Supplementary file 2 — Supplementary file3 (DOCX 17 KB) [file 392_2020_1784_MOESM2_ESM.docx]

**Supplemental Table 1. Baseline characteristics before propensity score matching**

|  | All  n=80 | Pascal  n=22 | MitraClip-XTR  n=58 | p value |
| --- | --- | --- | --- | --- |
| Age (year) | 78 ± 7 | 79 ± 5 | 78 ± 7 | 0.61 |
| Sex female, n (%) | 46 (58) | 14 (64) | 32 (55) | 0.62 |
| BMI (kg/m^2^) | 25.6 [21.8, 27.4] | 24.5 [20.8, 27.4] | 25.7 [22.2, 27.4] | 0.34 |
| Hypertension, n (%) | 68 (85) | 18 (82) | 50 (86) | 0.73 |
| Diabetes mellitus, n (%) | 22 (28) | 4 (18) | 18 (31) | 0.40 |
| COPD, n (%) | 20 (25) | 7 (32) | 13 (22) | 0.40 |
| Atrial fibrillation, n (%) | 75 (94) | 21 (96) | 54 (93) | 0.99 |
| Coronary artery disease, n (%) | 48 (60) | 13 (59) | 35 (60) | 0.99 |
| Prior cardiac surgery, n (%) | 51 (64) | 14 (64) | 37 (64) | 0.99 |
| Prior pacemaker/ICD/CRT implantation, n (%) | 25 (31) | 5 (23) | 20 (35) | 0.42 |
| NYHA III/IV, n (%) | 74 (93) | 21 (96) | 53 (91) | 0.99 |
| EuroSCORE II (%) | 8.4 [5.3, 12.6] | 7.8 [4.3, 12.1] | 8.8 [6.0, 12.9] | 0.38 |
| NT-pro-BNP (pg/ml) | 2153 [1253, 4220] | 1892 [1150, 3290] | 2287 [1323, 5342] | 0.17 |
| e-GFR (ml/min/1.73m^2^) | 46.9 [35.7, 64.9] | 46.8 [35.4, 64.9] | 47.4 [38.2, 64.5] | 0.99 |
| Total bilirubin (mg/dl) | 0.8 [0.5, 1.1] | 0.8 [0.5, 1.0] | 0.8 [0.6, 1.1] | 0.77 |
| AST (IU/L) | 28.0 [22.0, 35.0] | 24.0 [22.0, 32.0] | 30.0 [22.0, 36.0] | 0.16 |
| Hemoglobin (g/dl) | 12.0 [10.5, 12.9] | 11.9 [10.8, 13.0] | 12.0 [10.5, 12.9] | 0.83 |
| LVEF (%) | 56.9 [52.8, 62.0] | 57.0 [55.2, 63.9] | 56.1 [50.5, 60.9] | 0.13 |
| MR moderate to severe or more, n (%) | 13 (16) | 3 (14) | 10 (17) | 0.99 |
| TR massive or torrential, n (%) | 39 (49) | 16 (73) | 23 (40) | 0.01 |
| TR grade | 4.0 ± 0.9 | 4.3 ± 0.8 | 3.8 ± 0.9 | 0.02 |
| EROA (mm^2^) | 51.5 [36.8, 75.8] | 74.5 [51.5, 119.8] | 46.0 [34.0, 64.5] | <0.001 |
| Vena contracta (mm) | 8.2 [7.0, 10.0] | 9.6 [7.2, 11.8] | 8.1 [6.8, 10.0] | 0.16 |
| Regurgitant volume (ml) | 50.0 [40.6, 61.0] | 51.8 [43.8, 70.1] | 48.0 [39.0, 60.0] | 0.07 |
| TR jet location |  |  |  |  |
| Central or antero-septal commissure | 80 (100) | 22 (100) | 58 (100) | 0.99 |
| Postero-septal commissure | 62 (78) | 19 (86) | 43 (74) | 0.37 |
| Antero-posterior commissure | 15 (19) | 8 (36) | 7 (12) | 0.02 |
| Coaptation gap (mm) | 5.6 [3.2, 8.0] | 6.2 [3.3, 9.2] | 5.3 [3.2, 7.2] | 0.16 |
| Tricuspid annulus diameter (mm) | 45.0 [40.0, 52.0] | 45.0 [42.0, 49.0] | 45.0 [39.0, 52.0] | 0.49 |
| RA area (mm^2^) | 34.8 [26.3, 40.3] | 39.7 [35.4, 42.6] | 30.3 [25.3, 37.8] | 0.005 |
| RV diameter (mm) | 46.0 [31.0, 53.0] | 54.0 [46.3, 57.8] | 53.0 [47.0, 57.0] | 0.74 |
| RV diameter mid (mm) | 38.5 [33.0, 46.0] | 39.5 [35.3, 48.5] | 38.0 [33.0, 44.0] | 0.44 |
| TAPSE (mm) | 17.0 [14.0, 20.0] | 16.5 [12.5, 19.0] | 17.0 [14.8, 20.0] | 0.42 |

**Abbreviations**: BMI, body mass index; COPD, chronic obstructive pulmonary disease; CRT, cardiac resynchronization therapy; EROA, effective orifice regurgitant area; ICD, intracardiac defibrillator; GFR, glomerular filtration ratio; MR, mitral regurgitation; LVEF, left ventricular ejection fraction; NT-pro-BNP, NT-pro-brain natriuretic peptide; RA, right atrial; RV, right ventricular; TAPSE, tricuspid annular plane systolic excursion; TR, tricuspid regurgitation.
