# Supplementary figures and images for: PASCAL versus MitraClip-XTR edge-to-edge device for the treatment of tricuspid regurgitation: a propensity-matched analysis
Source: Clin Res Cardiol. 2020 Dec 12;110(3):451–9. doi: 10.1007/s00392-020-01784-w (PMC7907034; doi:10.1007/s00392-020-01784-w)

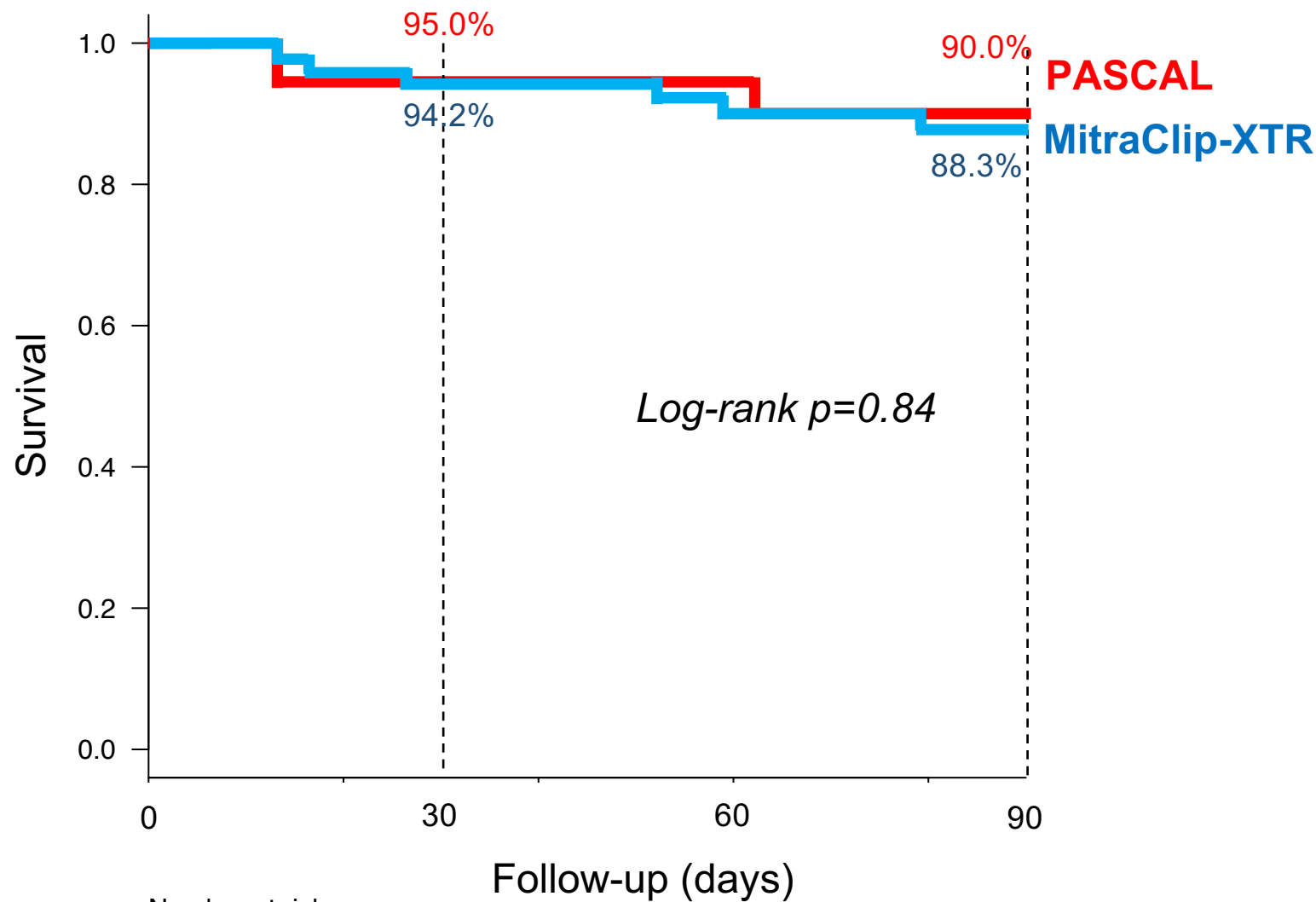

Number at risk

|                      |    |    |    |    |
|----------------------|----|----|----|----|
| <b>PASCAL</b>        | 22 | 20 | 20 | 19 |
| <b>MitraClip-XTR</b> | 58 | 51 | 48 | 43 |

Supplement: Supplementary file 3 — Supplemental Figure 1. Study population. During the study period, 80 patients were treated with the PASCAL or MitraClip-XTR systems for the treatment of TR. After propensity score matching, 44 patients (22 PASCAL vs. 22 MitraClip-XTR) were included into the present analysis [file 392_2020_1784_MOESM3_ESM.pdf]
