# Supplementary material for: PASCAL versus MitraClip-XTR edge-to-edge device for the treatment of tricuspid regurgitation: a propensity-matched analysis
Source: Clin Res Cardiol. 2020 Dec 12;110(3):451–9. doi: 10.1007/s00392-020-01784-w (PMC7907034; doi:10.1007/s00392-020-01784-w)
Supplement: Supplementary file 4 — Supplemental Figure 2. Survival analysis for entire cohort. There was no significant difference in 30-day and 3-month mortality between the PASCAL and MitraClip-XTR groups [file 392_2020_1784_MOESM4_ESM.pdf]

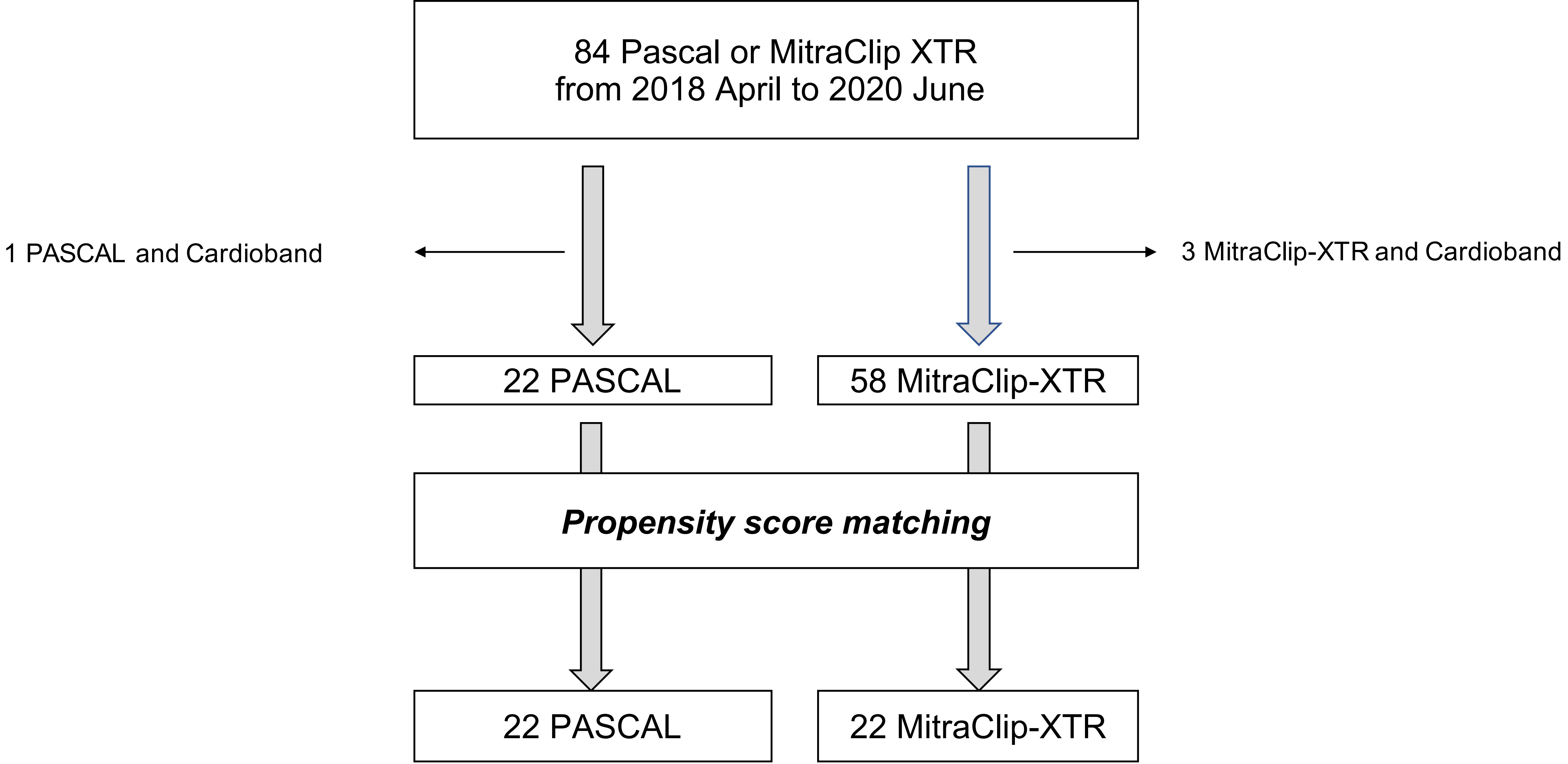
84 Pascal or MitraClip XTR
from 2018 April to 2020 June
1 PASCAL and Cardioband
3 MitraClip-XTR and Cardioband
22 PASCAL
58 MitraClip-XTR
Propensity score matching
22 PASCAL
22 MitraClip-XTR
